# Targeting protein–protein interactions to rescue Δf508-cftr: a novel corrector approach to treat cystic fibrosis

**DOI:** 10.1002/emmm.201303301

**Published:** 2013-08-27

**Authors:** Isabel Devesa, Gregorio Fernández-Ballester, Antonio Ferrer-Montiel

**Affiliations:** 1 Instituto de Biología Molecular y Celular, Universidad Miguel HernándezAlicante, Spain

**Keywords:** drug discovery, ion channel, lung disease, proteostasis, therapy

See related article in EMBO Molecular Medicine http://dx.doi.org/10.1002/emmm.201302699

Cystic fibrosis (CF) is one of the most prevalent life-threatening autosomal recessive disorders in the Western World, with an estimated incidence of 1 in 2000–3000 Caucasian newborns and a median age of survival of 40 years. The first report of CF as a distinct pathology was published in 1938 by Dorothy Andersen, a physician at the Babies & Children's Hospital of Columbia University, who differentiated its diagnosis from celiac disease in the pancreas (Andersen, 1938). This seminal description was followed by other contributions that linked CF to altered Cl^−^ permeability in epithelial cells in the gastrointestinal and hepato-billiary systems, respiratory tract, reproductive system and sweat glands (Kreindler, 2010); and culminated with the discovery of the gene responsible of the disease, referred to as the cystic fibrosis transmembrane regulator (CFTR) (Riordan et al, 1989). The CFTR is a cAMP-regulated chloride and bicarbonate channel that controls anion conductance in epithelial cells located in mucosal surfaces. Functional alteration of CFTR leads to the accumulation of mucous secretions that cannot be cleared favouring inflammation and infection. Cumulative evidence shows a strong correlation between the severity of the CF phenotype and the degree of CFTR dysfunction, being stronger for those patients where the channel function is absent (Kreindler, 2010).

The CF phenotype is caused by more than 1000 mutations of the CFTR gene including missense, such as R117H or G551D that significantly reduce channel activity, and nonsense like G542X, R553X or W1282X, which abrogate protein expression (Kreindler, 2010). However, the most prevalent mutation in CF patients (70%) is the in-frame deletion of Phenylalanine at position 508 (ΔF508-CFTR) (Kreindler, 2010; Rowe & Verkman, 2013). This deletion produces a partially unfolded CFTR protein that is retained in the endoplasmic reticulum and diverted to degradation by the proteosome, resulting in a virtually absent CFTR activity in CF patients (Lukacs & Verkman, 2012). In addition, the ΔF508-CFTR mutant is a dysfunctional channel that display abnormal channel gating characterized by a lower open probability than the wild type protein (Lukacs & Verkman, 2012). Accordingly, amelioration of this CF phenotype requires restoration of CFTR channel activity in epithelial cells by augmenting CFTR trafficking to the cell surface and by potentiating its channel gating.

» A noticeable weakness of discovered corrector molecules is the lack of convincing data on their mechanism of action which is pivotal to understand their limited clinical efficacy and critical to rationally develop the next generation of correctors with improved activity.«

Several therapeutic strategies have been used to reinstate or compensate for CFTR activity and improve patient symptomatology and quality of life (Kreindler, 2010). For nonsense mutants with premature termination codons in their sequence, the main approach has been centred in promoting their expression using ribosomal read-through molecules such as PTC124 (Ataluren, PTC Therapeutics), which is currently in Phase II clinical trials (Wilschanski et al, 2011). For missense and the ΔF508-CFTR mutants the therapeutic approaches used include increasing Na^+^ absorption, promoting the activation of Ca^2+^-dependent chloride channels (Denufusol, Inspire Pharmaceuticals), as well as the use of antimicrobials and anti-inflammatories (Hanrahan et al, 2013). However, most of these treatments are not aimed at correcting the cause of the disease and display side effects that limit their clinical utility. Consequently, there is an urgent necessity of developing novel, more efficacious and safe CF therapeutics.

In the past years, a great effort has been placed in understanding the biology and biophysics of missense and ΔF508-CFTR phenotypes with the aim of designing compounds tailored at correcting their dysfunction. These efforts have lead to search for small molecules that enhance the channel activity, known as channel potentiators; and, compounds which facilitate its trafficking to the plasma membrane, referred to as correctors; along with dual-acting molecules exhibiting both potentiator and corrector activities (Hanrahan et al, 2013; Rowe & Verkman, 2013). Large drug-like compound libraries, screened using high throughput assays aimed at identifying molecules that stimulate CFTR activity of missense mutations, have lead to the identification of potentiators such as VX-770 (Ivacaftor, Vertex Pharmaceuticals) that restore channel function *in vitro* and *in vivo* (Rowe & Verkman, 2013). Noteworthy, in a proof-of-concept clinical trial with 39 patients carrying the G551D mutation, Ivacaftor administered orally exhibited a within-subject improvement in CFTR markers and lung function, suggesting that potentiators may be a viable therapeutic approach for the treatment of at least some CF patients (Accurso et al, 2010). However, this clinical study failed to show statistical significance between the treated and placebo groups, plausibly because of the low number of patients used. Thus, a larger clinical study appears warranted given the partial positive outcome of this pilot assay.

Corrector compounds are very interesting proteostasis modulators directed at incrementing the maturation of partially misfolded ΔF508-CFTR protein, thus facilitating its trafficking to the cell membrane. Corrector molecules may target a plethora of proteins that modulate CFTR folding and its quality control system including chaperones, phosphodiesterases, PARPs and kinases. Notably, most known proteostasis modulators that increase the trafficking of ΔF508-CFTR to the plasma membrane (sildenafil, glafenine, genistein and curcumin) appear to be inhibitors of posttranslational modifications (Hanrahan et al, 2013). Regrettably, proof of concept clinical trials with glafenine and curcumin have failed to confirm therapeutic corrector activity in humans (Rowe & Verkman, 2013). Small molecules derived from screening large chemical libraries using cell-based assays are represented by the bithiazole corr-4a and compound VX-809 (Rowe & Verkman, 2013). A clinical trial in ΔF508-CFTR homozygous CF patients showed that VX-809, the most potent identified corrector, reduced sweat chloride as compared with placebo, although no improvement in lung function was observed (Clancy et al, 2012). Currently, VX-809 is being tested in combination with Ivacaftor to evaluate whether combination of correctors and potentiators may enhance CFTR activity in the clinics.

**Figure 1:**
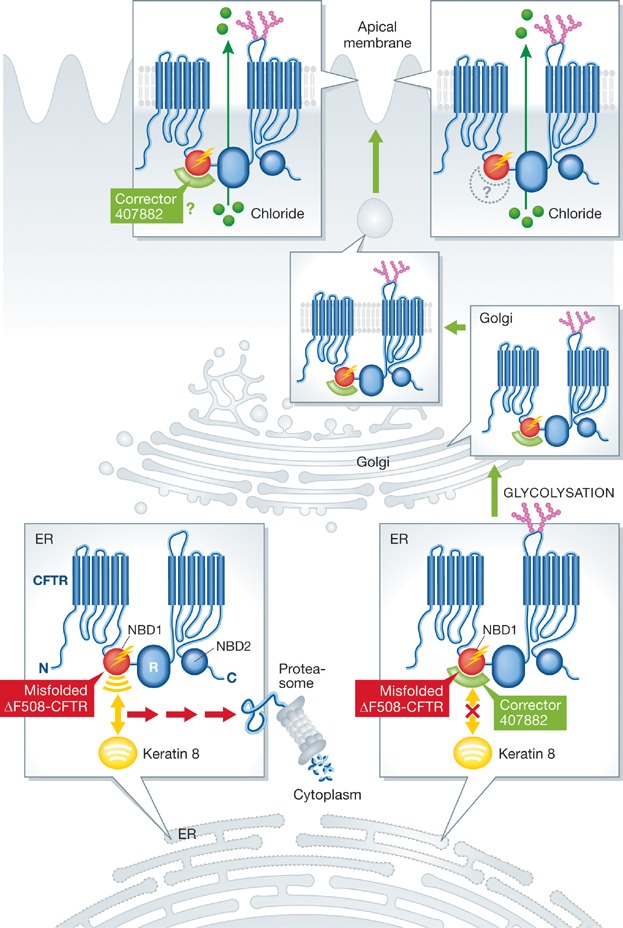
Corrector molecules increment the surface expression of ΔF508-CFTR channel Misfolded ΔF508-CFTR protein in the endoplasmatic reticulum (ER) interacts with housekeeping proteins such as keratin 8 and is primed for the degradation pathway that ends in the proteosome. Corrector 407882 binds to exposed surfaces in the partially misfolded NBD1, preventing the interaction with keratin 8, and facilitating the glycosylation of the protein and trafficking to the Golgi and the plasma membrane where it restores chloride permeability. A remaining question is whether corrector 407882 is required also for function, or it may be displaced by proteins interacting with the channel in the cell surface.

A noticeable weakness of discovered corrector molecules is the lack of convincing data on their mechanism of action which is pivotal to understand their limited clinical efficacy and critical to rationally develop the next generation of correctors with improved activity. Thus, there is an urgent necessity to develop proteostasis modulators with a well know site of action and enhanced activity. This is precisely the salient contribution of the paper by Odolczyk et al (2013) that reports the discovery of novel corrector compounds with a defined molecular and cellular mechanism that significantly improve CFTR activity *in vitro* and *in vivo*. Based on previous observations showing differences in the dynamic behaviour between the first nucleotide binding domain (NBD1) of ΔF508- and wild type-CFTR, the authors hypothesized the small molecules targeted to the unique conformational state of the ΔF508-CFTR NBD1 may prevent its interaction with housekeeping proteins that recognize unfolded proteins, thus averting its degradation and facilitating its trafficking to the plasma membrane. By using a structural-based virtual screening on the hydrophobic, solvent-exposed areas of the ΔF508-CFTR NBD1, they identified four new ΔF508-CFTR proteostasis modulators, and demonstrate that at least two of them, 407882 and 118208, preclude the interaction of the mutated protein with the housekeeping protein keratin 8. Interestingly, these correctors do not appear to stabilize the mutated and unfolded NBD1 domain implying a novel mechanism of action for CF medications. Furthermore, this study also provides a novel pharmacological target for treating CF patients, and paves the way to therapeutically validate and exploit other complexes that prevent ΔF508-CFTR trafficking to the cell surface (Fig 1).

» …the compounds discovered by Odolczyk et al (2013) evidently represent a novel family of CFTR correctors for CF drug intervention.«

The most promising corrector, 407882, is a water-soluble molecule that contains two phenylphosphinic groups. Its binding site appears to be located in pocket 2 of the NBD1 domain, where it is stabilized by both polar and hydrophobic interactions. Notably, the *in vitro* efficiency of 407882 appears to be significantly higher than that of corrector VX-809 and, in addition, it is capable of rescuing CFTR function in a cell-type independent manner. Although this compound has not been proven in humans yet, the observation that is able to restore the channel function to a significant degree in a mouse model of CF demonstrates an *in vivo* correcting activity, which should be the foundation for its clinical development. Of note, because corrector 407882 recognizes a specific surface environment located in the ΔF508-CFTR mutant channel, it could be anticipated that this modulator may display limited off side effects that, along with its water solubility, further enhance its therapeutic index.

Although there are yet questions that remain answered regarding their mechanism of action and clinical utility, the compounds discovered by Odolczyk et al (2013) evidently represent a novel family of CFTR correctors for CF drug intervention. Nonetheless, we should keep in mind that akin to other proteostasis modulators exhibiting significant *in vitro* correcting activity, this new class of correctors might not live up to clinical expectations. However, the combination of 407882 with potentiator molecules such as Ivacaftor may provide a feasible strategy to enhance the clinical effectiveness of both categories of therapeutics thus providing novel and useful medications for this prevalent fatal autosomal genetic disorder.
